# Annotations

**Published:** 1887-09-24

**Authors:** 


### ANNOTATIONS.

**Honest Yorkshire Farmers.** It is stated by the Analyst for Sheffield that butterine is purchased by farmers and incorporated with genuine butter. Everybody is accustomed to town people adulterating their goods, and there is an organised system of inspection conducted in defence of purchasers of town manipulated articles. Hitherto we have been in the habit of thinking that country produce at least was safe from sophistication. It was not that anyone really imagined country folk to be more honest than those of the town, because there are scoundrels everywhere, and if a man have a knavish disposition he will show it whether the temptation be great or small. But there has been a kind of feeling that farmers, especially Yorkshire farmers, had some decent degree of pride which prevented them from descending to wretched little arts for the sake of turning a dishonest shilling. That apparently is all a delusion, and in the neighbourhood of Sheffield some mean and disgraceful rascal has actually gone to the butterine shop, and purchased butterine to take back to his own dairy for the purpose of there manufacturing "genuine farmer's butter." For our part we should not have been sorry if the butterine had contained a charge of dynamite and had exploded as it was being mixed with the finest produce of the cow. A man like this farmer has too thick a hide to be reached by words, even if, as is unlikely, The Hospital should fall into his hands. But if the law were what it ought to be, he and all his like would be visited with such a punishment as would effectually deter imitators. The man who sells goods as pure, knowing them to be adulterated, is one of the basest scoundrels that modern society has to deal with, and well deserves to be taught the art of oakum-picking, and to practise it for a twelvemonth.

**"The Cow with the Iron Tail."** Country people enjoy a grim joke, and the pump from which the farmer draws the water he mixes with his milk is known among them as the "cow with the iron tail." Some of the Local Authorities in London appear to have much sympathy with the dilution of milk by the purveyors of that uncertain fluid. Perhaps they think the public stomach is so degenerated by high civilisation that it cannot digest the pure article as the cow supplies it; or is it that they are anxious to stamp out the present epidemic of scarlet fever by attenuating any dangerous virus the unmixed milk may possibly contain? The Vestry of St. Mary, Newington, according to the report of the Local Government Board, appears to think the Adulteration Acts quite superfluous. A dairyman in the neighbouring parish, who was fined, complained, and not unnaturally, that he was hardly dealt with, in that he only adulterated his milk to the same degree as his neighbour and rival, who was not fined at all, simply because he happened to live on the opposite side of the way. What is the reason of this excess of charity on the part of the authorities who are retained for the protection of the public? Is their chairman a dairyman, or does he take shares in some Limited Liability Pure Milk Company? It is satisfactory to find that the custom of adulterating milk is on the wane. Of a large number of samples examined, only 11.9 per cent. were found to have been tampered with. The authorities at the Local Government Board are of opinion that the fines imposed upon those who cheat are too small to be effectually deterrent, the conscienceless knaves being able to do much more than recoup themselves by the profit on the added water. As in the case of the "Butterine Farmers," we would recommend an acquaintance with the inside of a prison for those honest gentlemen.

**Wholesale Poisoning.** Man acknowledges one principle of morals in which he would be cordially agreed with by all the lower animals, lions, reptiles, and pigs alike, and that is the sacred duty of claiming and keeping all he can for himself. He does this in the mass as well as in detail. It is not only every *man* for himself and somebody who shall be nameless take the hindmost, but it is every town and every village and every hamlet for itself, and let whole populations be poisoned with sewage in their drinking water if they do not resist. Water is drawn from the Thames and the Lea high up towards the source of those rivers, and is supplied to the people of London for domestic use. Although it has been less impure in 1886 than in former years, it has been by no means ideally perfect. In the case of the Thames the sewage from towns containing together 70,000 people is still thrown into the river *above* the intake for the London water-supply. Those towns, with their 70,000 inhabitants, do not purify their sewage in any way before sending it down the Thames to flavour the liquid drunk by the people below. The idea is not at all nice, especially for those who neither filter nor boil their drinking water. What a very unpractical people we are! The five millions of London could eat up the 70,000 of Staines and the neighbouring towns at a single meal; and yet they permit them to foul and poison the water in this disgraceful way almost without remonstrance. Who knows how many lives are lost and homes desolated annually by this poisonous fluid? What is the use of rulers and Parliaments if they cannot protect a quarter of the whole population of England against such dangers as these. One longs for a despot well instructed in sanitary science, who would in one year sweep away all these disgusting and dangerous anomalies, and give us, if not pure air, at least pure water to drink.

